# Prolonged oral cannabinoid administration prevents neuroinflammation, lowers β-amyloid levels and improves cognitive performance in Tg APP 2576 mice

**DOI:** 10.1186/1742-2094-9-8

**Published:** 2012-01-16

**Authors:** Ana María Martín-Moreno, Begoña Brera, Carlos Spuch, Eva Carro, Luis García-García, Mercedes Delgado, Miguel A Pozo, Nadia G Innamorato, Antonio Cuadrado, María L de Ceballos

**Affiliations:** 1 Neurodenegeration Group, Dept. of Cellular, Molecular and Developmental Neurobiology, Instituto Cajal, CSIC, Doctor Arce, 37, Madrid, 28002, Spain; 2 Centre for Biomedical Research on Neurodegenerative Diseases (CIBERNED), Madrid, Spain; 3 Neuroscience Group, Research Institute Hospital 12 de Octubre, Av. de Córdoba s/n Madrid, 28041, Spain; 4 CAI de Cartografía Cerebral, Instituto Pluridisciplinar, UCM, Paseo Juan XXIII 1, Madrid, 28040, Spain; 5 PET Technology Institute, Manuel Bartolomé Cossio 10, Madrid, 28040, Spain; 6 Departamento de Bioquímica, Instituto de Investigaciones Biomédicas "Alberto Sols"UAM-CSIC, Facultad de Medicina, Universidad Autónoma de Madrid, Arturo Duperier 4, Madrid, 28029, Spain; 7 Current Address: MD Anderson Cancer Center, Arturo Soria 270, 28033 Madrid, Spain; 8 Current Address: Department of Pathology and Neuropathology, University Hospital of Vigo (CHUVI), Vigo, Spain

**Keywords:** Alzheimer's disease, β-amyloid peptide, cannabinoids, glial activation, interleukin 6, anti-inflammatories, tumor necrosis factor-α

## Abstract

**Background:**

Alzheimer's disease (AD) brain shows an ongoing inflammatory condition and non-steroidal anti-inflammatories diminish the risk of suffering the neurologic disease. Cannabinoids are neuroprotective and anti-inflammatory agents with therapeutic potential.

**Methods:**

We have studied the effects of prolonged oral administration of transgenic amyloid precursor protein (APP) mice with two pharmacologically different cannabinoids (WIN 55,212-2 and JWH-133, 0.2 mg/kg/day in the drinking water during 4 months) on inflammatory and cognitive parameters, and on ^18^F-fluoro-deoxyglucose (^18^FDG) uptake by positron emission tomography (PET).

**Results:**

Novel object recognition was significantly reduced in 11 month old Tg APP mice and 4 month administration of JWH was able to normalize this cognitive deficit, although WIN was ineffective. Wild type mice cognitive performance was unaltered by cannabinoid administration. Tg APP mice showed decreased ^18^FDG uptake in hippocampus and cortical regions, which was counteracted by oral JWH treatment. Hippocampal GFAP immunoreactivity and cortical protein expression was unaffected by genotype or treatment. In contrast, the density of Iba1 positive microglia was increased in Tg APP mice, and normalized following JWH chronic treatment. Both cannabinoids were effective at reducing the enhancement of COX-2 protein levels and TNF-α mRNA expression found in the AD model. Increased cortical β-amyloid (Aβ) levels were significantly reduced in the mouse model by both cannabinoids. Noteworthy both cannabinoids enhanced Aβ transport across choroid plexus cells *in vitro*.

**Conclusions:**

In summary we have shown that chronically administered cannabinoid showed marked beneficial effects concomitant with inflammation reduction and increased Aβ clearance.

## Background

Alzheimer's disease (AD) is the major cause of dementia. The cognitive impairment is associated with the degeneration of particular subsets of neurons in regions involved in learning and memory processes. In addition another invariant feature of AD is neuroinflammation, considered a consequence of glial activation and reflected as astrogliosis and microglial activation, in particular around senile plaques, one of the pathological hallmarks of the disease, along neurofibrillary tangles. Indeed, lots of inflammatory parameters are found in AD brains [1,2]. Once initiated the inflammatory process it may contribute independently to neural dysfunction and cell death, establishing a self-perpetuating vicious cycle by which inflammation induces further neurodegeneration. The recognition of inflammation as an important component in the disease led to the discovery that prolonged treatment with non-steroidal anti-inflammatories (NSAIDS) had beneficial effects for AD. Indeed, several prospective works have shown that this kind of treatment markedly reduced the risk of suffering the neurologic condition, delayed its onset, ameliorated the symptomatic severity and slowed cognitive decline [3-5]. However their administration to already demented patients may be ineffective, suggesting the importance of early administration or, alternatively, the existence of additional targets of NSAIDs, besides cycloxygenase inhibition. Nevertheless, other compounds with anti-inflammatory activity may be disease modifying drugs, which may delay onset or slow its progression, in contrast with the present AD palliative treatment.

Cannabinoids, whether plant derived, synthetic or endocannabinoids, interact with two well characterized cannabinoid receptors, CB_1 _and CB_2 _[6,7]. In addition, some cannabinoids may interact with other receptors, such as the TRPV_1 _receptor or the orphan receptor GPR55 [8,9]. The CB_1 _receptor is widely distributed, with a particularly high expression in brain, which contrasts with the limited expression of the CB_2 _receptor, which is characteristic of immune organs and cells [10]. In fact, while CB_1 _receptors are expressed by all types of cells in the brain (neurons and glial cells), CB_2 _are mainly localized in microglial cells [6,9-11], the resident immune cell of the brain.

We and others have proposed cannabinoids as preventive treatment for AD [12-14], based on their neuroprotective [15,16] and anti-inflammatory effects [11,17,18]. Indeed, cannabinoids are able to decrease the release of cytokines and nitric oxide in cultured microglial cells induced by lipopolysacharide [19,20] and Aβ addition [12,21]. In several *in vitro *studies cannabidiol (CBD), the major non-psychotropic constituent of cannabis, has shown to be neuroprotective against β-amyloid (Aβ) addition to cultured cells. This action was a consequence of reduction of oxidative stress and blockade of apoptosis [22], tau-phosphorylation inhibition through the Wnt/β-catenin pathway [23] and decreased iNOS expression and nitrite generation [24]. *In vivo *experiments have shown that several cannabinoids were effective at preventing Alzheimer's disease related changes. In a previous work we have reported that synthetic cannabinoids, such as WIN55,212-2 and JWH-133, prevented the cognitive impairment, glial activation and neuronal marker loss in β-amyloid injected rats [12]. Enhancement of endocannabinoid levels by subchronic uptake inhibition reversed the increase in inflammatory parameters, such as COX-2, TNF-α and IL-1, in Aβ-injected mice, although cognitive impairment was only prevented in early treated mice [25]. Further, we have recently reported that CBD and WIN55,212-2 (WIN) inhibited both glial activation and cognitive deficits, as judged in the water maze test, and by a mechanism involving decreasing microglial activation, as shown in cultured microglial cells [20].

So far the possible effects of cannabinoids have not been studied in a genetic AD model, which mimics the amyloidosis and neuroinflammation [26,27] that occurs in the neurologic condition, in the absence of overt neurodegeneration [28]. Therefore in this work we have addressed the question whether cannabinoid agonists would ameliorate the cognitive deficits, neuroinflammation and altered Aβ levels in this AD model. To that end we have used WIN 55,212-2 (WIN), a mixed CB_1_/CB_2 _agonist [6] and JWH-133 (JWH) as selective CB_2 _agonist [29]. The CB_2 _selective agonist is devoid of psychoactivity, an advantage for its clinical endorsement, however we reasoned that a low dose of WIN may be free of psychoactive effects as well. The drugs were administered in drinking water [30] for a prolonged period, 4 months, to mimic a possible clinical setting. Given that cannabinoid treatment may be preventive, but not curative, administration of drugs was started at 7 months of age, when no cognitive dysfunction or plaque deposition exists, and it was prolonged for 4 months (eg until 11 months of age). We report here that such a treatment ameliorates cognitive performance, decreases neuroinflammation and Aβ levels, likely by increasing its transport to the periphery.

## Results

### Effect of cannabinoid oral treatment on cognitive impairment and glucose uptake by PET

In a previous work we assessed the effects of cannabinoids following intraventricular administration concomitant to Aβ injection for 7 days to rats [12]. This type of administration is not feasible in the Clinic, and is restricted to very few disease conditions. Other authors have used oral administration of 0.1 mg/kg/day tetra-hydrocannabinol (THC) effectively [30]. Therefore, in this work we have chosen to administer cannabinoids in the drinking water to investigate their effects in the genetic model of AD. Given that we aimed a preventive treatment, it was initiated at 7 months of age when they do not exhibit Aβ deposition in plaques [28].

**Figure 1:**
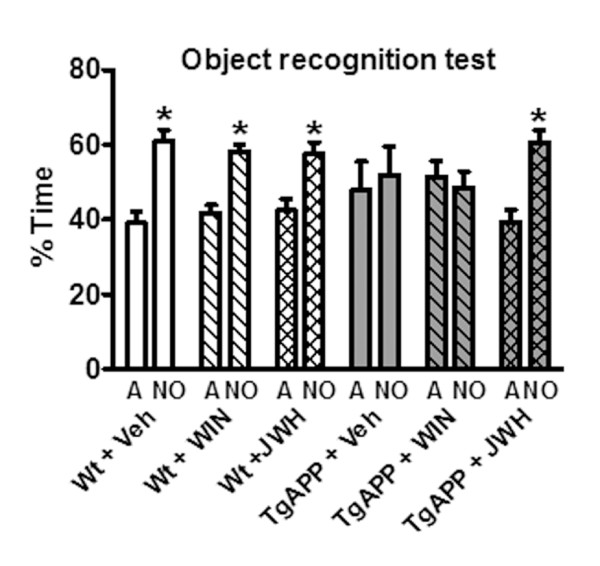
**JWH oral administration rescued the cognitive impairment of TgAPP**. Cannabinoid agonists did not alter the discrimination of the novel object (NO) compared to the familar one (A) of wt mice. Tg APP failed to distinguish the NO, like those treated with WIN, but JWH treatment restored normal discrimination. Animals were treated with vehicle or cannabinoids (0.2 mg/kg) in the drinking water for 4 months, starting at 7 months of age. Time spent exploring either objects was expressed as percentage of the total exploration time. Results are mean ± SEM (5-7 mice/group). *p < 0.05 vs familiar object (ANOVA followed by Student's t test).

First we examined whether continuous oral treatment with a low dose (0.2 mg/kg/day for 4 months) of cannabinoids was able to rescue the cognitive impairment of Tg APP eleven months old mice in the novel object recognition test. Exploration activity was monitored during the training trial and all the animals displayed similar attention to the two identical objects. Wild type mice treated with vehicle were able to discriminate between the familiar object (Figure 1) and a novel object, exploring the latter for a longer time. WIN and JWH did not affect this behavior in wild type mice, even after prolonged treatment. As expected Tg APP mice did not distinguish both objects, showing cognitive impairment. In contrast Tg APP mice treated with the CB_2 _selective cannabinoid JWH spent more time exploring the novel object, although WIN was ineffective at that respect.

**Figure 2:**
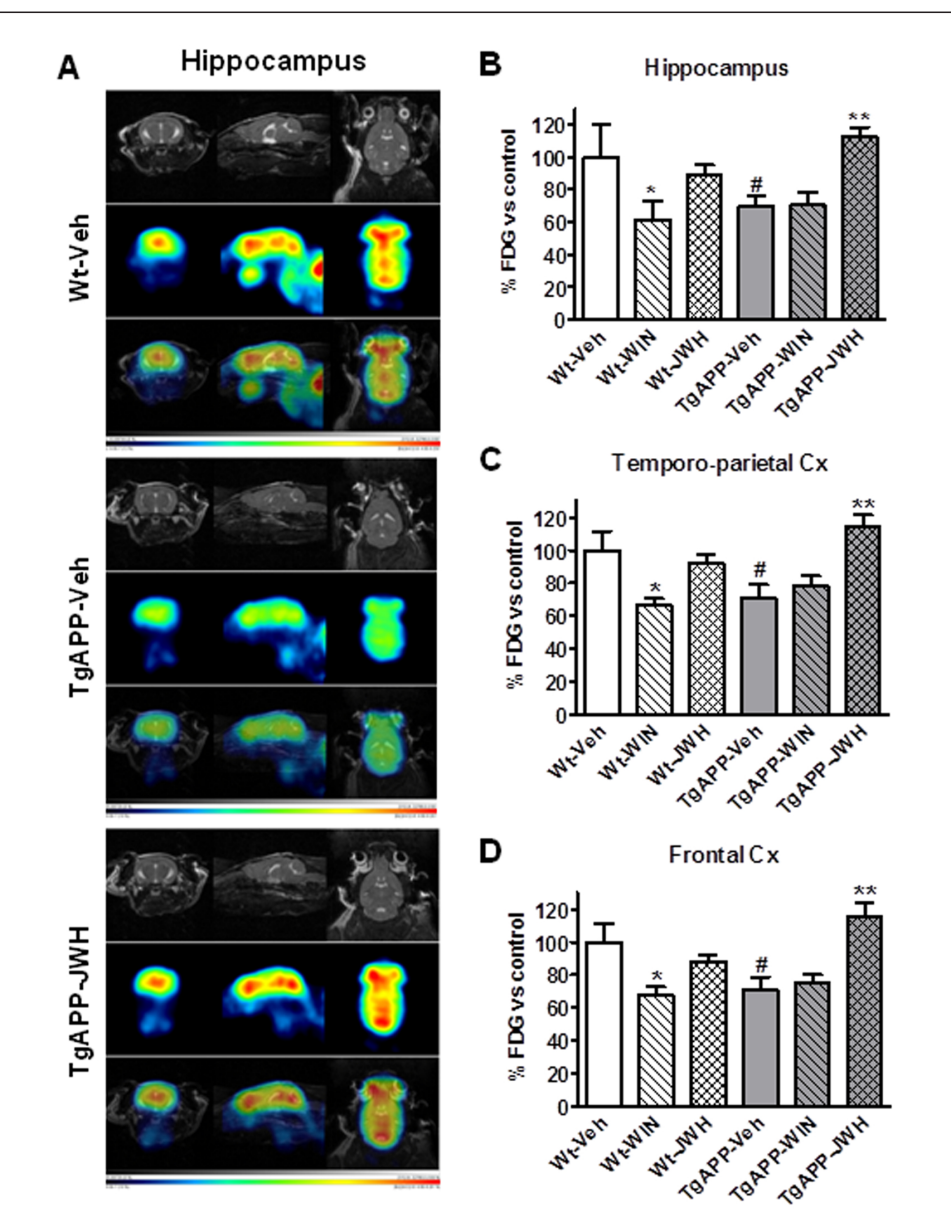
**JWH oral administration rescued the decreased ^18^F-DG uptake in TgAPP assessed by PET**. A: representative images [MR, ^18^F-DG uptake and merged] in hippocampus of wild type (Wt) and Tg APP vehicle treated mice. Continuous oral treatment of wild type mice with WIN decreased uptake in all regions studied (B: hippocampus; C: Frontal cortex; D: Temporo-parietal cortex). Tg APP showed decreased uptake which was normalized by JWH oral treatment (A-D). Animals were treated with vehicle or cannabinoids (0.2 mg/kg) in the drinking water for 4 months, starting at 7 months of age. Results are mean ± SEM (n = 4 mice/group) of the ratio of average radiactivity in a given region of interest (ROI) by the radiactivity in cerebellum (Cb) expressed as percentage. *p < 0.05, ** p < 0.01 vs vehicle treated mice; #p < 0.05 vs wild type-vehicle mice (ANOVA followed by Student's t test).

^18^FDG uptake is significantly decreased in areas involved in learning and memory both in AD patients and in subjects showing mild cognitive impairment [31]. Moreover it is considered to be an early marker of the neurologic disease. Prolonged WIN treatment reduced the uptake in wild type mice (Figure 2 B, C, D), while JWH was without effect. As expected 12 month Tg APP vehicle treated mice had reduced ^18^FDG uptake (25-30% depending on the region of interest; Figure 2 A, B, C, D). JWH continuous administration fully reversed the reduction in glucose uptake in Tg APP mice (Figure 2 A, B, C, D), which was slightly higher than that of wild type mice (approximately 15%), while WIN was ineffective on that respect. Therefore JWH, the CB_2 _selective agonist, was capable of improving cognitive impairment and of reversing the reduction in ^18^FDG uptake in cortical areas and hippocampus of Tg APP.

### Cannabinoids reduce microglial activation and inflammatory parameters in Tg APP mice cerebral cortex

**Figure 3:**
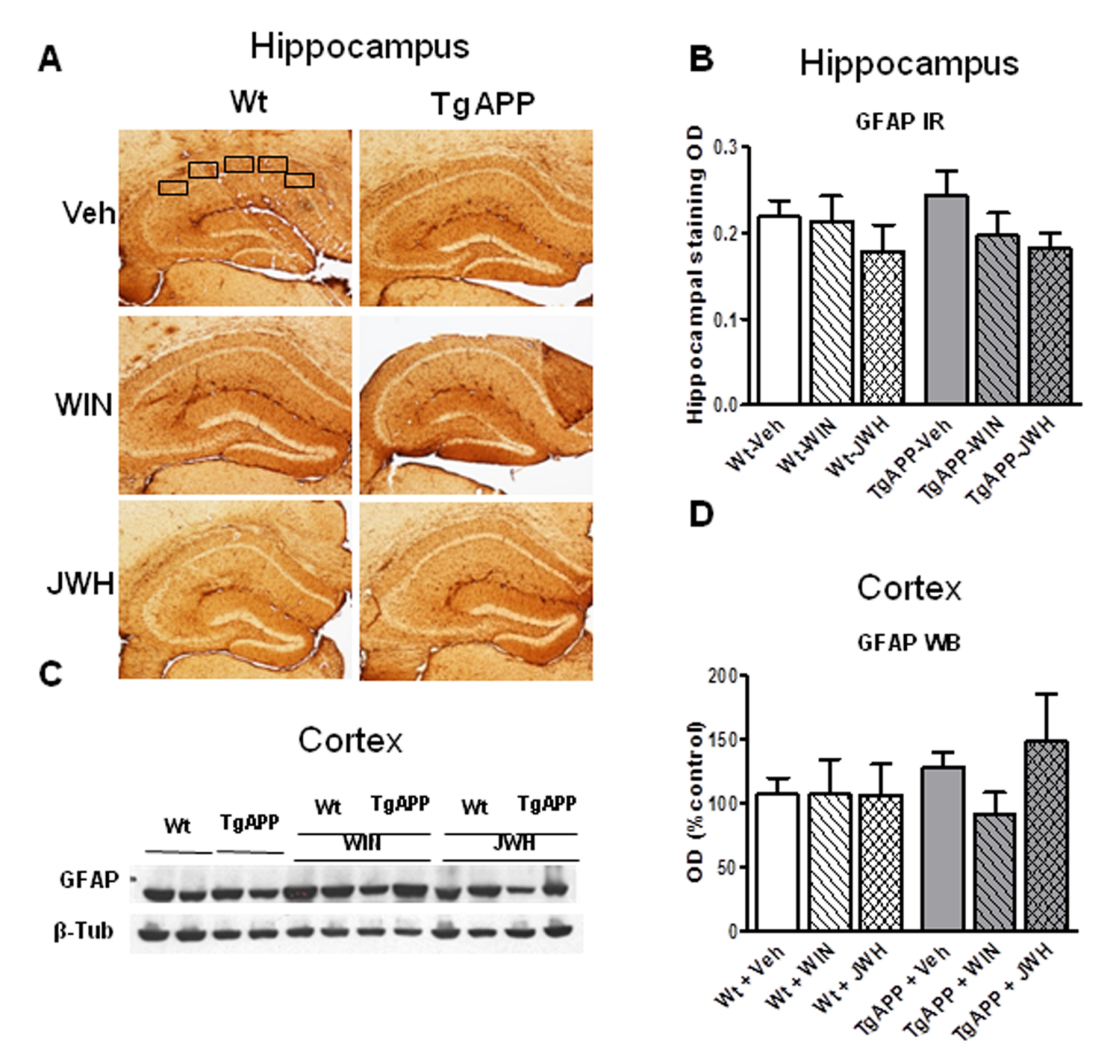
**Cannabinoid oral administration did not alter GFAP immunostaining or protein expression**. GFAP hippocampal immunostaining (IR) or cerebral cortical protein expression was similar in all groups, irrespective of genotype or treatment. A: representative IR in hippocampus from wild type and Tg APP mice treated with vehicle or cannabinoids. The area assessed is shown (5 hippocampal areas) B: Optical density (OD) was measured by densitometry. C: representative GFAP *Western blot *(WB) and D: optical density (OD) in cortical samples. Animals were treated with vehicle or cannabinoids (0.2 mg/kg) in the drinking water for 4 months, starting at 7 months of age. Results are mean ± SEM (n = 6-7 mice/group).

**Figure 4:**
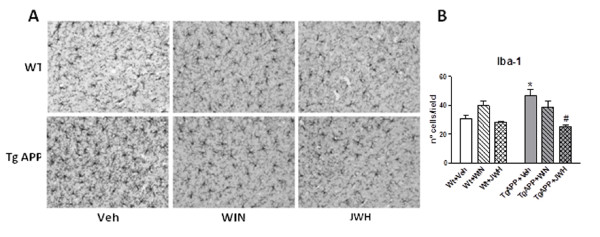
**Microglial cell density was increased in Tg APP and decreased by continuous JWH oral treatment**. A: representative Iba-1 immunostaining in cerebral cortex following the different treatments. B: microglial cortical cell density. Animals were treated with vehicle or cannabinoids (0.2 mg/kg) in the drinking water for 4 months, starting at 7 months of age. Results are mean ± SEM (n = 6-7 mice/group). #p < 0.05 vs wild type vehicle treated mice; * p < 0.05 vs Tg APP vehicle treated mice (ANOVA followed by Student's t test).

Neuroinflammation is reflected in AD and its transgenic models brain as astrogliosis and microglial activation, particularly surrounding senile plaques [26,28]. GFAP immunohistochemistry showed similar staining patterns in hippocampus in the different groups studied (Figure 3 A and 3B), irrespective of the genotype or the treatment received. Similarly GFAP protein levels, as assessed by Western blotting, were maintained across the different groups (Figure 3 C and 3D). Microglial cell staining and density was assessed by using ionized Ca^2+^-binding adaptor molecule-1 (Iba-1) antibody. The antibody labeled resting microglial cells in wild type mice, characterized by the fine tortuous branching, which was unaffected by either of the cannabinoids used in the oral chronic treatment (Figure 4, A). Tg APP vehicle treated mice showed an increase (53%) in Iba-1 positive microglial cells compared with wild type mice (Figure 4, A and 4B). While continuous WIN treatment did not alter this increased density, the CB_2 _selective agonist JWH decreased cell density so that cell number accounted 83% of that of wild type mice (Figure 4 B). These results indicate that at this age Tg APP mice do not show astrogliosis, but they had microglial activation, which was effectively counteracted by JWH oral treatment.

**Figure 5:**
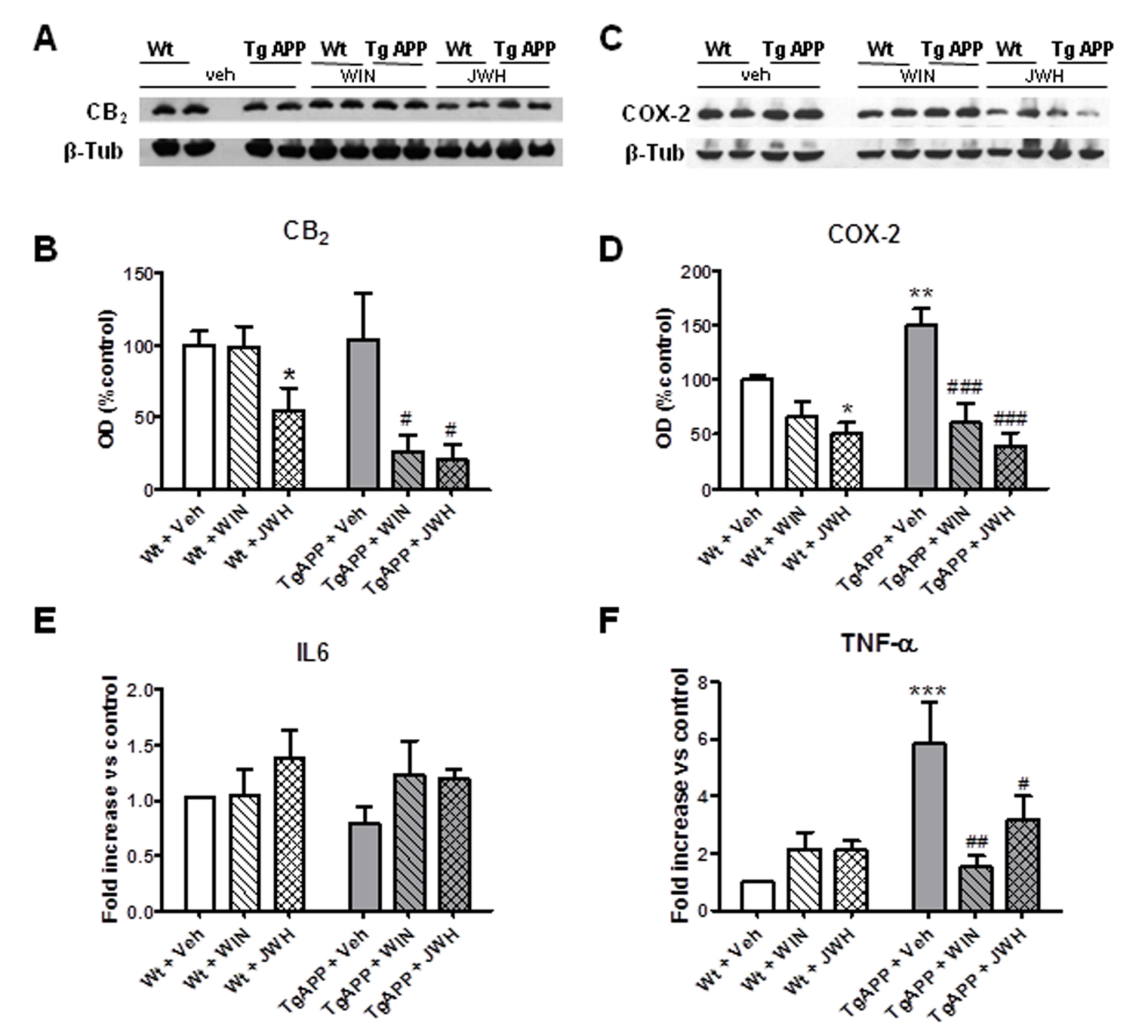
**Cannabinoid oral treatment decreased inflammatory parameters of Tg APP mice**. A, B: Cannabinoids decreased CB_2 _protein levels by *Western blotting *in Tg APP. C, D: Cannabinoids counteracted the increase in COX-2 protein levels by *Western blotting*. E: IL6 mRNA expression (qRT-PCR) was similar in all groups studied. F: Cannabinoids blocked the increased TNF-α mRNA expression (qRT-PCR) in Tg APP. Animals were treated with vehicle or cannabinoids (0.2 mg/kg) in the drinking water for 4 months, starting at 7 months of age. Results are mean ± SEM (n = 6-7 mice/group) *p < 0.05, ** p < 0.01 vs vehicle treated mice; #p < 0.05 vs wild type mice vehicle treated mice (ANOVA followed by Student's t test).

Recent works have shown increased CB_2 _microglial expression following a lesion or in a neurodegeneration context [32], including AD brain [13,33]. We also addressed this issue by means of double immuno-fluorescence. We revealed CB_2 _expression in Iba-1 microglial cells (additional file 1 A1-3) in quinolinic acid injected striatum (additional file 2) and not in the contralateral striatum (additional file 1, B1-3), as expected. However there was no evident CB_2 _staining in any region examined of Tg APP mice (additional file 1, C1-3). Regarding CB_2 _protein expression Tg APP mice had similar levels compared to wild type mice which also received vehicle (Figure 5 A and 5B). JWH administration, but not WIN, to wild type mice reduced (45%) CB_2 _protein levels (Figure 5, B). Both WIN and JWH significantly decreased CB_2 _protein (Figure 5, B) in the AD model, which attained very low levels (around a 75%-80% reduction).

We used Western analysis or qRT-PCR to assess possible changes in inflammatory parameters in cerebral cortex (Figure 5). JWH, but not WIN, decreased COX-2 expression in wild type mice (Figure 5 C and 5D). This parameter was markedly increased a 49% in Tg APP mice (Figure 5 D). Noteworthy, both cannabinoids effectively reversed COX-2 protein enhancement, reaching even lower levels than in wild type mice (40% and 60% respectively vs. wild type vehicle treated mice).

Different cytokines have been shown to be increased in AD brain and in its genetic model. IL-6 mRNA expression was similar across the different groups (Figure 5, E). As expected cortical TNF-α mRNA expression was dramatically increased (around 6 fold vs wild type vehicle treated mice; Figure 5 F). The cannabinoid agonists used in this study prevented this increase, although this cytokine expression continued to be higher than that of wild type mice (Figure 5, F).

Taken together these results demonstrate that continuous cannabinoid administration has anti-inflammatory activity given that different mediators were effectively counteracted in Tg APP.

### Cannabinoids oral administration decreases β-amyloid levels and increases its transport through choroid plexus monolayers

**Figure 6:**
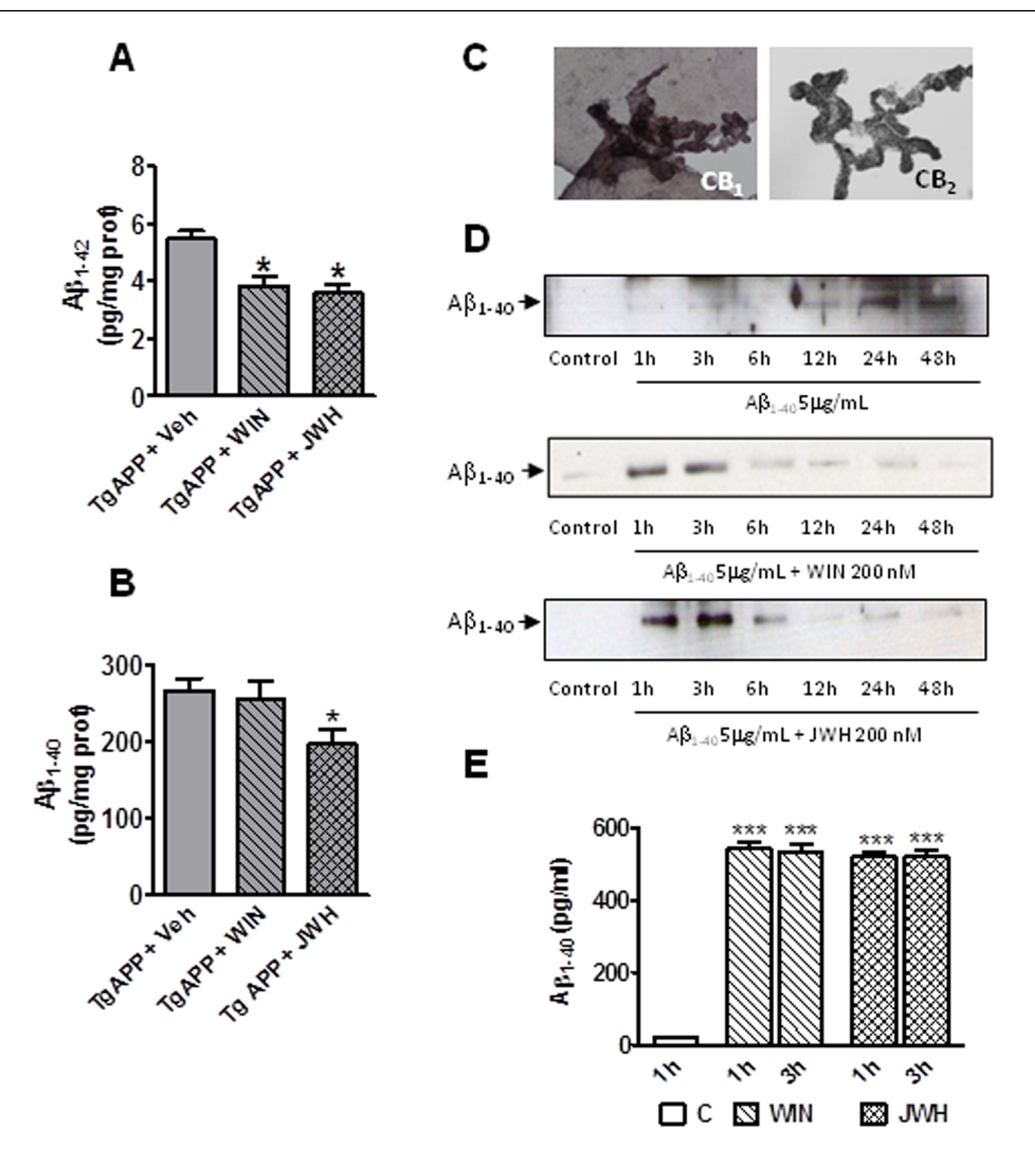
**Cannabinoids decreased Aβ levels of Tg APP mice and increased transport through choroid plexus cell monolayer**. A: Both cannabinoids significantly decreased Aβ_1-42 _cortical levels. B: JWH decreased Aβ_1-40 _cortical levels in Tg APP mice. Animals were treated with vehicle or cannabinoids (0.2 mg/kg) in the drinking water for 4 months, starting at 7 months of age. Results are mean ± SEM (n = 6-7 mice/group) C: rat choroid plexus express both CB_1 _and CB_2 _receptors. D: time-course of Aβ_1-40 _transport through choroid plexus cells. Both cannabinoids (200 nM) reduced the transport time compared to untreated cultures (representative *Western blot*). E: Cannabinoids enhanced Aβ_1-40 _transport through choroid plexus cells at 1 and 3 h after addition vs untreated control cultures (mean ± SEM, n = 4). E: *p < 0.05, *** p < 0.01 vs vehicle treated mice or cultures (Student's t test).

The Tg APP model parallels the amyloidosis of the neurologic condition. Eleven month old Tg APP mice have no detectable insoluble (formic acid extracted) Aβ levels by ELISA. As expected no soluble human transgenic Aβ levels were detected in wild type mice, while Tg APP mice had high levels of Aβ_1-40 _(Figure 6, A) and detectable levels of the more amyloidogenic Aβ_1-42 _fragment (Figure 6, B). JWH decreased by 27% the levels of Aβ_1-40_, and a 30% similar reduction in Aβ_1-42 _was found following the administration of either WIN or JWH (Figure 6, A and 6B).

To determine the mechanism by which both cannabinoids reduce Aβ levels first we examined the effect of both compounds on Aβ release from cultured APP/PS1 glioma cells. There was no difference in the presence or the absence of drugs (data not shown) suggesting that neither the release nor the synthesis of Aβ was altered. Next we wondered whether the cannabinoids would reduce Aβ levels by enhancing its clearance, a therapeutic strategy intensively studied for AD treatment. Therefore we assessed Aβ transport across choroid plexus monolayers *in vitro*. Since there are contradictory reports on the presence of CB_1 _receptors in choroid plexus [34,35] first we performed stainings with CB_1 _and CB_2 _antibodies. We revealed the presence of both receptors in rat choroid plexus (Figure 6, C). Then we examined the possible time course of the transport of Aβ_1-40 _(5 μg/ml) added to the upper compartment, by measuring the amount of peptide in the lower compartment by Western blotting at different time points (Figure 6 D). In the absence of any drug the peptide was recovered at 12 h, and at longer times (24 and 48 h), after its addition to the upper compartment. However, the transport was much quicker in the presence of WIN or JWH (200 nM) and Aβ was mainly transported at shorter times (1-3 h; Figure 6 D). To quantify this transport we took advantage of ELISA assays and assessed peptide levels at 1 and 3 h after the addition of Aβ to the cell cultures (Figure 6 E). WIN and JWH promoted the same peptide transport both at 1 and 3 h, while almost no detectable Aβ crossed the choroid plexus cell monolayer in their absence (Figure 6 E). In summary, prolonged oral administration of cannabinoids effectively reduced Aβ levels in Tg APP brain, likely due to increased peptide clearance through the blood- or the CSF-barrier.

### Effect of prolonged oral treatment with cannabinoids on GSK3-β levels

**Figure 7:**
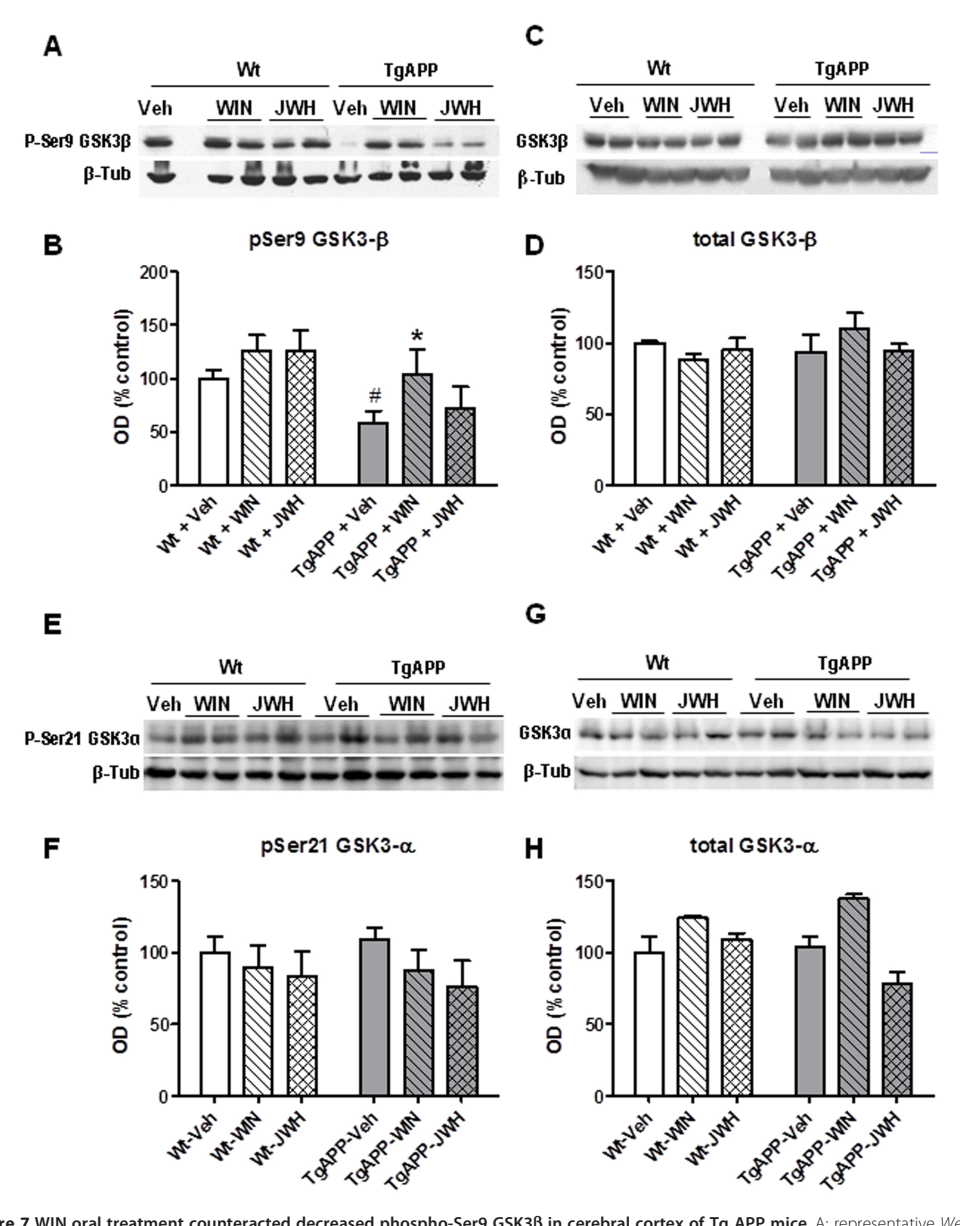
**WIN oral treatment counteracted decreased phospho-Ser9 GSK3β in cerebral cortex of Tg APP mice**. A: representative *Western blot *of p-Ser9 GSK3β. B: optical density (OD). C: total-GSK3β. D: optical density (OD). E: representative *Western blot *of p-Ser21 GSK3α. F: optical density (OD). G: total-GSK3α in cerebral cortex. H: optical density (OD). Wild type (Wt) and Tg APP mice were treated with vehicle or cannabinoids (0.2 mg/kg) in the drinking water for 4 months, starting at 7 months of age. Results are mean ± SEM (n = 6-7 mice/group) *p < 0.05 vs wt-veh mice, # p < 0.05 vs Tg APP-vehicle treated mice; #p < 0.05 vs wild type vehicle treated mice (ANOVA followed by Student's t test).

AD is characterized by the existence of increased tau phosphorylation, mainly by the action of GSK3-β, which is deregulated. We assessed by Western blotting total and pSer9-GSK3-β (inactive form) levels in cerebral cortex, from Wt and Tg APP mice following the different treatments. Cannabinoid agonist treatment did not change p-Ser9 GSK3-β protein levels in wild type mice. In contrast, there was a reduction (around 40%) in p-GSK3-β in the AD model which was normalized by WIN administration, but not JWH (28% decrease vs wild type vehicle treated mice). Total GSK3-β protein levels remained largely unchanged. Similarly no changes in the levels of either p-Ser21 GSK3-α or total GSK3-α protein levels were observed (Figure 7). Therefore, the pathological pSer9-GSK3-β activity was normalized by the CB_1_/CB_2 _mixed agonist WIN.

## Discussion

In this work we describe several beneficial effects of the prolonged oral treatment with two cannabinoid agonists with different pharmacological profiles. Both cannabinoids were effective at decreasing the inflammatory parameters and Aβ levels. However, it was the CB_2 _selective agonist JWH that was able to prevent cognitive deficits and glucose metabolism reduction.

Our results differ from a recent work [36] reporting variable effects on water maze performance and fear conditioning, no changes in Aβ levels or plaque burden following subchronic treatment with the CB_1_/CB_2 _mixed agonist HU-210. Methodological differences may account for the results, since they used male and female double APP23/PS1 transgenic mice, at 4 (young) or 6-9 weeks of age, and a very low dose of the cannabinoid agonist (10 or 50 μg/kg) injected twice daily [14].

CB_1 _activation induces psychoactive effects. In fact mixed CB_1_/CB_2 _and CB_1 _selective agonists after acute administration decrease motor activity and impair learning and memory [37-39], although at higher doses than the one we used in this study (0.2 mg/kg/day). However, chronic administration of those drugs induces tolerance to their acute effects in different behavioural tests [40,41]. Therefore, the fact that WIN did not affect learning in the novel object recognition test in wild type mice might suggest tolerance after prolonged administration. Similarly WIN was without effect on recognition memory of Tg APP mice. In contrast, CB_2 _selective agonists, such as JWH, are devoid of psychoactivity after acute administration and does not alter motor activity [[42], Martín-Moreno et al., in preparation] after systemic administration or object recognition memory following its intrahippocampal injection [43]. After prolonged administration to wild type mice JWH neither altered learning nor memory in the present work. Interestingly this compound effectively counteracted the cognitive impairment of Tg APP mice.

The brain is the organ with the highest glucose consumption, which is believed to be coupled to neural activity. The reduction in brain glucose uptake has been repeatedly demonstrated in AD patients [31,44], in particular in regions involved in memory, and it is highly correlated with cognitive deficits. Prolonged oral WIN administration reduced glucose uptake, as measured by PET ^18^F-DG, in wild type mice in cortical regions and hippocampus. This result may be deleterious, in spite of the observation of no memory impairment in the cognitive test selected. In fact, previous autoradiographic work has reported either normal ^3^H-DG uptake in hippocampus or a decrease, depending on the dose of WIN [45] acutely administered. Decreased ^18^F-DG uptake in Tg APP in the present work is in essential agreement with previous reports, either by autoradiographic techniques [46] or PET [47]. Notably JWH administration for 4 months did not alter glucose uptake in wild type mice, while it completely abrogated the reduction observed in Tg APP. Brain activity in general, and cognition in particular rely on glucose metabolism [47], therefore the effects of the CB_2 _agonist on both glucose utilization and recognition memory are of therapeutic interest.

Inflammation has several drawbacks including learning and memory impairment [48], in particular during ageing [49,50]. The compounds under study behaved as anti-inflammatory agents, in agreement with previous reports [11,12,20,25,49-51]. Microglial activation, but not astrogliosis, was observed in 11 month old Tg APP mice. Previous works have shown prominent reactive astrogliosis and increased GFAP expression in transgenic mice, that appears to be age-dependent and related with disease progression [26,28,52], albeit being restricted to plaques. The lack of astrogliosis and changes in GFAP expression may be explained by the absence of plaques in the mice model at this age. This result is in agreement with the detection of GFAP mRNA, as assessed by non-radioactive *in situ *hybridization, in reactive astrocytes in close proximity with Aβ plaques at 14 months of age, but not before, when plaques were absent [53]. Indeed we found no plaques with glial associated cells, although there was a significant enhancement in microglial cell density in Tg APP mice. Continuous JWH treatment for 4 months normalized this parameter, but WIN was ineffective. In different contexts (eg lesions) both astrocytes and microglia could be engaged in inflammation. However in light of those results we can ascribe the increase in inflammatory mediators to microglial activation, given that there was no overt astrogliosis. COX-2 protein levels and TNF-α mRNA expression is increased in AD and its transgenic model [54,55]. Both cannabinoids significantly decreased COX-2 and TNF-α, as expected, since both compounds share CB_2 _receptor activation, as shown by the down-regulation of its expression in Tg APP mice. It should be noted that although CB_2 _receptors could be expressed by some neurons [56] they are mainly expressed by microglial cells [6,9-11], and are involved in the modulation of several inflammatory mediators [12,19-21,25]. We did expect an increase in CB_2 _receptor expression in the transgenic model given that Aβ addition to microglial cultures enhance it [21]. However, in Tg APP mice we did not observe CB_2 _co-localization with Iba-1, which contrasts with the microglial co-expression in AD brain [13,33]. We neither found an increase by Western blotting, in agreement with our previous results in AD patients or Aβ-injected rats [12]. This supports the notion that at this age there is an ongoing glial activation of low magnitude in Tg APP and that cannabinoids down-modulate this response.

Aβ removal is considered a therapeutic strategy in AD, promoted either by vaccination [57,58] or by enhancing its clearance towards the periphery [59,60]. One of the most interesting findings of the present work is the Aβ lowering ability of both cannabinoids, which we report for the first time. Prolonged oral JWH treatment decreased Aβ_1-40 _levels in brain and both cannabinoids decreased the more amyloidogenic fragment, Aβ_1-42_. Given that the drugs did not alter Aβ release we speculated that APP cleavage was not altered, and therefore studied whether cannabinoids changed the peptide transport *in vitro*. Rat choroid plexus expressed CB_1_, in agreement with [34], and also CB_2 _receptor protein, making feasible their activation by the drugs under study. Cannabinoids favored Aβ transport, that was mainly observed at shorter times (1-3 h) compared to control experiments. This interesting effect merits further study.

At variance with those effects oral treatment with WIN, but not JWH, normalized the levels of GSK3-β in Tg APP mice. Neurofibrillary tangles (NFTs), resulting from an abnormal phosphorylation of microtubule-associated tau proteins, represent a key pathological hallmark of Alzheimer's brain. GSK3-β is the kinase mainly responsible for tau hyperphosphorylation, therefore inhibiting its activity is considered of therapeutic interest. The effect of WIN after prolonged oral administration is in accordance with reports showing a CB_1 _dependent increase in GSK3-β phosphorylation in cultured cells [61], in brain after acute cannabinoid agonist administration [62], and with a reduction in tau phosphorylation [23]. Neurofibrillary tangles are intraneuronal elements and neurons are in general devoid of CB_2 _receptors. Therefore the effects of WIN on GSK3-β, which were not mimicked by the CB_2 _selective agonist JWH, might be explained by its interaction with CB_1 _receptors in neurons.

Given that our previous work had shown that cannabinoids were preventive against the Aβ effects, both *in vitro *and *in vivo *[12,20] we decided to start the continuous oral treatment at 7 months of age. At this time Tg APP mice do not have plaques and show normal learning and memory compared to wild type mice. Nevertheless, the treatment ended at 11 months of age when Tg APP begin to show memory disruption. According to our results the prolonged drug treatment decreased microglial activation of Tg APP mice along several inflammatory mediators, which were increased. However, ageing alters microglial responsiveness (eg to Aβ production and deposition), which is highly dynamic and context dependent [63]. Therefore a potential caveat of our results is that they may not be applicable to aged pathological microglia as occurs in severe AD. However, a preventive treatment at very early stages of the disease may be feasible and beneficial as has been shown with the anti-inflammatory trifusal, both in amnestic mild cognitively impaired patients [64] and in Tg APP mice [65].

Over the last decade important findings on the involvement of the endocannabinoid system in AD has been gathered. Indeed, in AD brain there is increased expression of CB_2 _receptors in microglia and of fatty acid amide hydrolase, the enzyme responsible for anandamide degradation, in astrocytes around plaques [13]. However CB_1 _localization is markedly altered and its protein expression and functionality diminished [12]. Furthermore, molecular rearrangements in different endocannabinoid system elements suggest that 2-AG signaling is increased, possibly contributing to synaptic failure in AD [66], while anandamide levels are decreased and are inversely correlated with Aβ levels [67]. Interestingly, the CB_2 _receptor expression has been reported to be increased both in the brain of AD patients [68] and in peripheral blood, where a significant correlation was found with the dementia score [69]. Finally, a CB_2 _PET radiotracer is accumulated in brains showing neuroinflammation (eg LPS injected and Tg APP/PS1 mice; [70]). These latter results suggest the importance of CB_2 _receptor as a biomarker of the neurologic disease, but also as a therapeutic target. CB_2 _receptor increased expression in AD appears to be a consequence of microglia activation, but more importantly they render microglia susceptible to cannabinoid modulation, decreasing the generation cytotoxic molecules and inhibiting microglial activation, while promoting its migratory activity [10,11,20].

## Conclusions

In summary, cannabinoid agonists, in particular CB_2 _selective agonists, interfere with several interconnected events of importance in the pathophysiology of AD. These compounds by directly interacting with cannabinoid receptors, in particular CB_2_, decrease microglial activation thereby reducing inflammation and its consequences (eg cognitive deficits). At the same time they may indirectly have beneficial effects on microglial activation (eg decrease cytokine release) by lowering brain Aβ levels.

## Methods

### Animals and treatments

Tg APP transgenic mice were obtained via heterozygous breeding of mice expressing the 695 aa long isoform of the human APP containing a double mutation Lys 670-Asn, Met 671-Leu [52] (swedish mutation) under transcriptional control of the hamster prion promoter on a C57/BL6 breeding background. Male Tg APP and wild type littermates, used as controls, were 7 months age at the beginning of the experiments. Mice were group-housed (4-5 animals per cage) with a 12:12 h light/dark cycle and with *ad libitum *access to food and water. All of the experiments were performed according to ethical regulations on the use and welfare of experimental animals of the European Union and the Spanish Ministry of Agriculture, and the procedures were approved by the bioethical committee of the CSIC.

WIN 55,212-2 (WIN) and JWH-133 (JWH) were administered in the drinking water at a dose of 0.2 mg/kg/day using ethanol (0.1%) as vehicle. The amount of water drank by the animals was assessed every other day and the treatment was adjusted to their weight. There was no difference in the body weight or the ingested water between groups, all along the experiment, discarding a possible reinforcing effect of cannabinoids.

Animals were sacrificed by cervical dislocation followed by decapitation at 11 months of age. The brain was saggitally divided. One hemisphere was rapidly dissected on a cold plate, frozen on dry ice and stored at -80°C until assayed. The other hemisphere was immersion fixed in PF 4% in PB 0.1 M for 24 h, cryoprotected in sucrose 15% (24 h) and 30% (24 h) in PB, snap frozen in hexane (-60°C) and stored at -20°C until cut with a sliding microtome.

### Novel object recognition test

The arena measured 40 × 40 cm, surrounded by 30-cm-high perimeter black walls, that was located in an isolated room that was novel to the animals. The floor of the arena was covered with used sawdust. The arena was monitored by a video-camera located above the arena. The procedure consisted in three visits to the arena in subsequent days. The first day mice were placed into the empty arena for 15 min (habituation). The second day they were allowed to explore two identical objects during two 10 min trials 5 min apart (training). On the third day (test), one of the objects was changed by a novel one, with different shape and color, and the mice explored the arena for 10 min. Data collection was carried out by the Ethovision software (Noldus, The Netherlands) and exploring was defined as "directing the nose at a distance equal to or less than 3 cm from the object or touching it with the nose". The time spent exploring the familiar object and the new object was expressed as a percentage. Objects were cleaned before every exposure with acetic acid 0.1% to prevent any olfactory clues. Experiments were performed at the same time of the day (9.00-14.00 h).

### ^18^Fdeoxyglucose (18FDG) Positron Emission Tomography (PET)

Fasted mice were anesthetized with isofluorane (2%) and injected (ip) with ^18^FDG (11,1 MBq or 300 μCi/200 μl saline, PET Technologic Institute, Madrid). Thirty min later ^18^FDG uptake images of each mouse were acquired for 30 min by PET imaging (Albira PET, 8 detectors, Gem-Imaging, Spain; [71]). The regions of interest were previously delineated in magnetic resonance (MR) T2-weighted images (Bruker Biospin, Germany) of each animal. Quantification of the metabolic activity was performed by co-registering the PET images of the brains to their own MR image as described by [72]. In our case, the field of view (FOV) of the PET scanner is 80 × 80 × 40 mm and the number of pixels of the reconstructed tomographic image is 160 × 160 × 80 pixels, being the voxel size 0.5 mm^3^. Co-registration of the PET image to the MRI, leads to a reduction of the pixel size to 0.2 mm^3^, given the trilinear interpolation done by the PMOD software (PMOD Technologies, version 2.9, Switzerland). The ^18^FDG uptake of the different brain areas were normalized to the ^18^FDG uptake in the cerebellum (considered as reference region).

### Immunohistochemistry and image analysis

Immunostaining was performed on floating sections (35 μm) as described previously [12]. In brief, following several washes with PBS, the endogenous peroxidase was blocked (3% hydrogen peroxide in methanol), washed again, and incubated for 90 min in PB containing 0.2% Triton × 100 and 10% normal goat serum. Sections were incubated with the different antibodies in PB containing 0.2% Triton × 100 and 1% normal goat serum overnight at 4°C. Dilutions of antibodies were as follows: anti-GFAP (1:1500, Sigma); anti-Iba-1 (1:1000, WAKO) (additional file 2). Development was conducted by the ABC method (Pierce, Rockford, IL), and immunoreactivity was visualized by 3,3-diaminobenzidine oxidation as chromogen, with or without nickel enhancement. Images were acquired with Zeiss Axiocam high resolution digital color camera, using the same settings and the segmentation parameters (MCID software, InterFocus Imaging, UK) were constant for a given marker and experiment. The mean value for each animal per region results from the analysis of 5-6 sections.

### Aβ levels

Aβ measurements were performed by two ELISA kits, one for each fragment (Aβ1-40 and Aβ1-42), from Biosource following the manufacturer instructions. The samples were sonicated (5 sec) in 10 vol of protein lysis buffer containing protease inhibitors (see Western blotting for details). The lysate was centrifuged (18,000 × g, for 10 min at 4°C). The supernatant was considered soluble and the pellets were further extracted by sonicating with formic acid and centrifuged. Prior to the Elisa the unsoluble samples were diluted 3 times with Tris 1 M, pH 10, while the soluble samples were diluted 5 times in Elisa buffer. The results were expressed as pg/mg of protein measured by the Bradford method, using BSA as standard.

### Aβ transport

A double-chamber choroid plexus epithelial cell culture system mimicking the blood-cerebrospinal fluid (CSF) interface was used for *in vitro *studies, as previously described [59]. After seeding, the cells were incubated for 24 h and thereafter Aβ_1-40 _(5 μg/ml) was added to the lower chamber in the absence or presence of the cannabinoid agonists (500 nM). At different time points the medium was collected from the upper chamber, and the Aβ_1-40 _content was determined by immunoblotting. In other experiments Aβ_1-40 _levels were assessed by selective Elisa kits (Biosource) as described above.

### Western blotting

Western blot was performed as described previously [61]. In brief, tissues were sonicated in lysis buffer, samples were centrifuged at high speed for 10 min, and supernatants were collected. Total protein was assessed by the Bio-Rad (Hercules, CA) protein assay. An aliquot of each sample (40 μg of protein) was separated by SDS-PAGE (10%), and proteins were transferred from the gels onto nitrocellulose membranes. The blots were blocked with 1% defatted dry milk for 1 h at room temperature and incubated overnight at 4°C with the following antibodies: anti-GFAP (1:10.000, DAKO), anti-CB_2 _(1:10.000, Affinity Bioreagents); anti-COX2 (1:100, Abcam); anti-p-GSK3β (1:5000, BD); anti-GSK3β (1:1.500, Cell Signaling) (additional file 2). Finally, samples were subjected to enhanced chemiluminescence and densitometric analysis. Band densitometric analysis was performed by Quantity One quantitation software (version 5.0; Bio-Rad) from film exposures; the background was subtracted, and the optical density percentage was obtained considering 100% the control samples within the same film. Tubulin was used as loading control. Every membrane contained samples from each treatment group, for comparison purposes.

The specificity of the CB_2 _antibodies used (additional file 2) was assessed by preincubating with the human antigenic peptide (2 μ/ml overnight at 4°C under agitation), as stated in additional file 2, before its use in Western blotting, which resulted in blockade of the immunoreactive signal (see additional file 3).

### Analysis of mRNA levels by quantitative real-time PCR

Total RNA from cortex was extracted using TRIzol reagent according to the manufacturer's instructions (Invitrogen). To avoid interference with potential genomic DNA amplification, we treated 1 μg of total RNA with 1 μl DNAse I (Invitrogen) plus 1 μl of 10× Buffer (Invitrogen). The samples were incubated at RT for 15 min. EDTA (25 mM) was added to the mixture and the samples were incubated at 65°C for 15 min to heat inactivate the DNAse I. For cDNA synthesis a total of 1 μg of RNA from the different samples were reverse-transcribed for 75 min at 42°C using 5 U of avian myeloblastosis virus reverse transcriptase (Promega) in the presence of 20 U of RNasin (Promega). The real-time PCR reaction was performed in 25 μl using the fluorescent dye SYBR Green Master mix (Applied Biosystems) and a mixture of 5 pmol of reverse and forward primers. The primers used were for TNF-α forward primer 5' CATCTTCTCAAAATTCGAGTGACAA 3' and reverse primer 5' TGGGAGTAGACAAGGTACAACCC 3' and for IL-6 forward primer 5' GAGGATACCACTCCCAACAGACC 3' and reverse primer 5' AAGTGCATCATCGTTGTTCATACA 3'. Quantification was performed on an ABI PRISM 7900 sequence detection system (Applied Biosystems). PCR cycles proceeded as follows: initial denaturation for 10 min at 95°C, then 40 cycles of denaturation (15 sec, 95°C), annealing (30 sec, 60°C), and extension (30 sec, 60°C). The melting-curve analysis showed the specificity of the amplifications. Threshold cycle, which inversely correlates with the target mRNA level, was measured as the cycle number at which the reporter fluorescent emission appears above the background threshold (data not shown). Data analysis is based on the ΔCT method with normalization of raw data to a house-keeping gene (β-actin). All of the PCRs were performed in triplicate.

### Statistical analysis

Statistical significance analysis was assessed by using one-way or two-way analysis of variance (ANOVA) followed by unpaired Student's t test (version 5.0, Prism software, GraphPad, USA). A value of p < 0.05 was considered significant.

## Abbreviations

Aβ: β-amyloid peptide; AD: Alzheimer's disease; COX-2: cycloxygenase; GSK3-β: glycogen synthase kinase 3-β; IL6: interleukin 6; TNF-α: tumor necrosis factor-α

## Competing interests

The authors declare that they have no competing interests.

## Authors' contributions

AMM-M carried out the mice treatment, the behavioural experiments, Western blotting, ELISA studies ex vivo, the molecular genetic studies, and helped to draft the manuscript. BB and MLC performed the immunohistochemical studies. NI and AC assessed qPCR experiments and Western blotting. LGG, MD and MAP designed, carried out and analyzed the PET studies. CS and EC designed and conducted the transport experiments in choroid plexus cell monolayers, including the ELISAs assays. MLC conceived of the study, participated in its design and coordination and completed the manuscript. All authors read and approved the final manuscript.

## Supplementary Material

Additional file 1 **Co-localization of CB_2 _and Iba-1 inmmunostaining in quinolinic acid injected striatum**. Iba-1 positive microglia in striatum also express CB_2 _receptor protein ipsilateral to the toxin injection, but was absent in the contralateral (unlesioned) striatum. Tg APP microglial cells are devoid of CB_2 _immunostaining. Click here for file

Additional file 2 **Additional methods**. (quinolinic acid striatal lesion and blockade of CB_2 _antibodies with CB2 blocking peptide) and additional file 1, which includes the antibodies, commercial source and dilution, used in immunohistochemistry and *Western blotting*. Click here for file

Additional file 3 **Blockade of CB_2 _antibodies 744 and 746 with CB_2 _antigenic peptide**. The CB_2 _peptide completely blocked immunostaining. Click here for file
