# Supplementary figures and images for: Prolonged oral cannabinoid administration prevents neuroinflammation, lowers β-amyloid levels and improves cognitive performance in Tg APP 2576 mice
Source: J Neuroinflammation. 2012 Jan 16;9:8. doi: 10.1186/1742-2094-9-8 (PMC3292807; doi:10.1186/1742-2094-9-8)

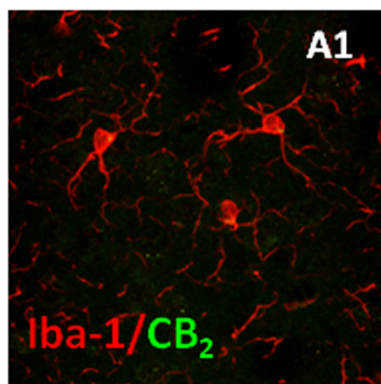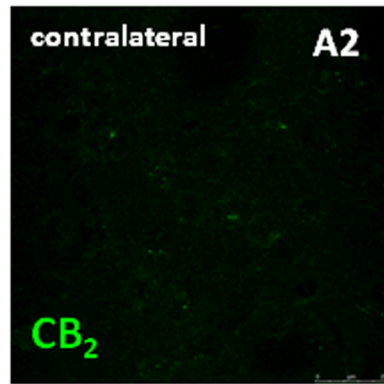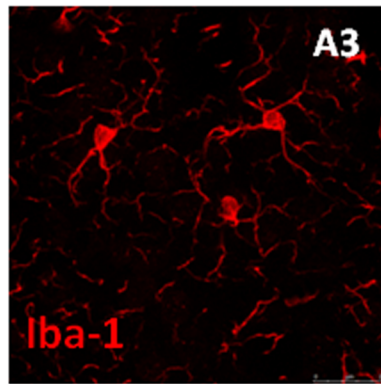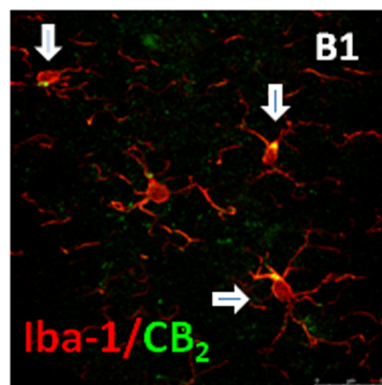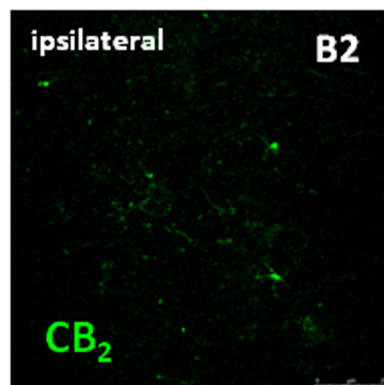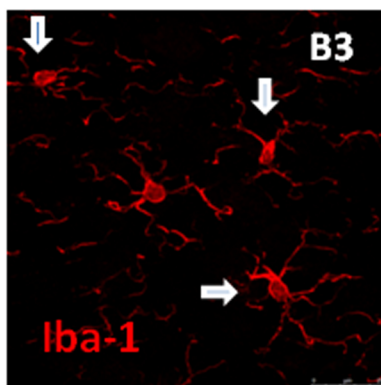

Quinolinic acid injected striatum

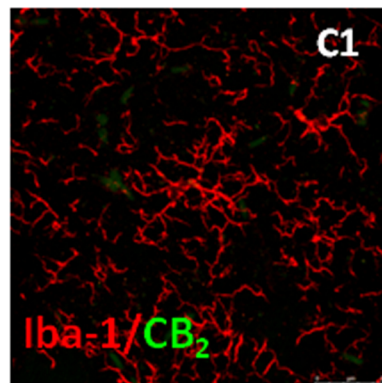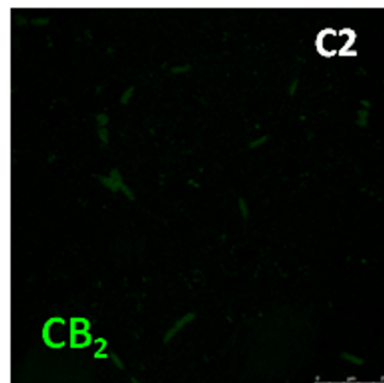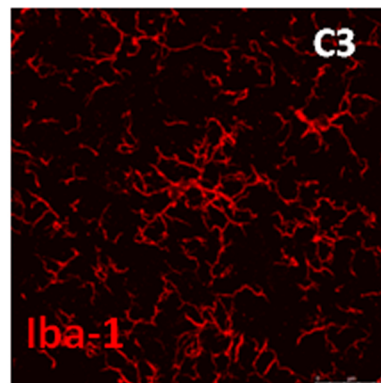

Tg APP cerebral cortex

Supplement: Additional file 1 — Co-localization of CB2 and Iba-1 inmmunostaining in quinolinic acid injected striatum. Iba-1 positive microglia in striatum also express CB2 receptor protein ipsilateral to the toxin injection, but was absent in the contralateral (unlesioned) striatum. Tg APP microglial cells are devoid of CB2 immunostaining. [file 1742-2094-9-8-S1.PDF]

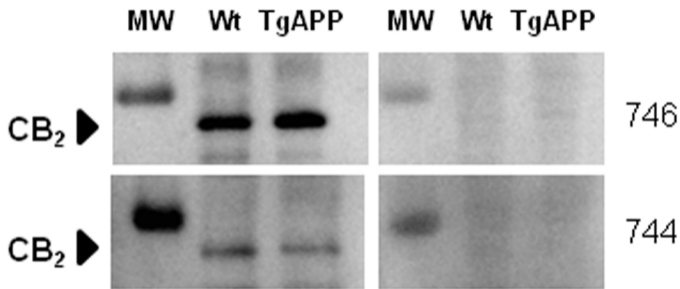

Supplement: Additional file 3 — Blockade of CB2 antibodies 744 and 746 with CB2 antigenic peptide. The CB2 peptide completely blocked immunostaining. [file 1742-2094-9-8-S3.PDF]
