# Supplementary material for: Prolonged oral cannabinoid administration prevents neuroinflammation, lowers β-amyloid levels and improves cognitive performance in Tg APP 2576 mice
Source: J Neuroinflammation. 2012 Jan 16;9:8. doi: 10.1186/1742-2094-9-8 (PMC3292807; doi:10.1186/1742-2094-9-8)
Supplement: Additional file 2 — Additional methods. (quinolinic acid striatal lesion and blockade of CB2 antibodies with CB2 blocking peptide) and additional file 1, which includes the antibodies, commercial source and dilution, used in immunohistochemistry and Western blotting. [file 1742-2094-9-8-S2.DOC]

**Additional files.**

**Striatal quinolic acid lesion**

Excitotoxicity was induced in adult C57/Bl6 mice by intrastriatal quinolic acid

(15 nmol ) injection at the following coordinates: +0.6 posterior to bregma,+1.85 medio-lateral and –2.7 dorso-ventral to dura. Glial activation was investigated at 7 days post-injection. Sections were kindly given by Dr. I Galve-Roperh (Palazuelos et al., 2009) and were used as positive control of Iba-1 and CB_2_ co-localization.

**Double CB_2_ and Iba-1 immunofluorescence staining**

For double immunofluorescence with anti-Iba-1 and anti-CB_2_ antibodies, the Fab fragment blocking system (Jackson Immunoresearch, Newmarket, UK) was employed following the manufacturer's instructions and the appropriate controls. In brief, after extensive washing of the sections (PBS, 6 x 15 min) they were blocked in PB containing 0.2 % Triton X 100 and 10% normal goat serum, for 90 min, followed by incubation in PB containing 0.2 % Triton X 100 and 1% normal goat serum, for 15 min. In this latter buffer the anti-CB_2_ antibody (1:500) was added and the sections were incubated for 2 x overnight at 4ºC. After extensive washing, sections were incubated at room temperature in the dark with FITC coupled secondary antibody Frab fragment (1:200) for 2 hours, followed by extensive washing. The sections were then incubated in Iba-1 antibody (1:1000) overnight at 4ºC, extensively washed, and incubated with anti-rabbit Alexa 647 for 2 hours, washed and mounted. Then, selected areas double-labeled were analyzed and photographed using a Leica TCS 4D confocal microscope.

**Preabsorption of CB_2_ antibodies with antigenic peptide**

The two different polyclonal antibodies against CB_2_ receptor protein were preincubated with the antigenic peptide prior to Western blot analysis. To this aim we used the synthetic peptide from the human CB_2_ sequence between the N-terminus and the first transmembrane domain, with the following amino acid sequence N-P-M-K-D-Y-M-I-L-S-G-P-Q-K (Cayman Chemical, USA). The antibodies were incubated with a final concentration of 2 µg/ml of the peptide, in PB 0.1 M, overnight at 4ºC under agitation and used in the incubation of the membranes after PAGE electrophoresis.

**Table 1 Antibodies used in the study**

Marker Host Dilution Source

CB_1_ rabbit 1: 600 K.Mackie

CB_2_ rabbit 1: 10,000 (WB) Affinity Bioreagents

(PAI-746)

CB_2_ rabbit 1: 500 (IHC) Affinity Bioreagents

(PAI-744)

COX-2 rabbit 1: 100 (WB) Abcam

GFAP mouse 1:1,500 (IHC) Sigma

GFAP rabbit 1: 10,000 (WB) Dako

p-GSK3-α (Ser21) rabbit 1: 1,000 (WB) Cell Signalling

GSK3-α rabbit 1: 2,000 (WB) Cell Signalling

p-GSK3-β (Ser9) rabbit 1: 5,000 (WB) Cell Signalling

GSK3-β mouse 1: 1,500 (WB) BD Transduction Labs.

Iba-1 rabbit 1: 1,000 (IHC) Wako

Antibodies used in either inmmunohistochemistry (IHC) or Western blotting (WB) are shown, along the host species, dilution and source.

**Legend to additional file 1.**

**Co-localization of CB_2_ and Iba-1 inmmunostaining in quinolinic acid injected striatum.** Iba-1 (red) positive microglia in striatum also express CB_2_ (green) receptor protein ipsilateral to the toxin injection (white arrows B1), but was absent in the contralateral (unlesioned) striatum (A1). Tg APP microglial cells are devoid of CB_2_ immunostaining (C1). A3, B3 and C3 depicts Iba-1 staining, A2, B2 and C2 CB_2_ staining and A1, B1 and C1 merged images obtained by laser confocal microscopy. All images shown were acquired at the same magnification. Bar in A1 25 µm.

**Legend to additional file 2.**

**Blockade of CB_2_ antibodies 744 and 746 with CB_2_ antigenic peptide.** Following overnight preabsorption of CB_2_ antibodies (1:5000) in the presence of 2 µg/ml of human CB_2_ peptide the membranes were incubated overnight and developed. MW: molecular weight marker, Wt: cerebral cortex lysate from a wild type-vehicle treated mice; TgAPP: cerebral cortical lysate from a TgAPP-vehicle treated mice. The CB_2_ peptide completely blocked immunostaining.
